# Domestic Cat Hepadnavirus: Molecular Epidemiology and Phylogeny in Cats in Hong Kong

**DOI:** 10.3390/v15010150

**Published:** 2023-01-03

**Authors:** Paolo Capozza, Maura Carrai, Yan Ru Choi, Thomas Tu, Omid Nekouei, Gianvito Lanave, Vito Martella, Julia A. Beatty, Vanessa R. Barrs

**Affiliations:** 1 Department of Veterinary Medicine, University of Bari Aldo Moro, 70010 Valenzano, Italy; 2 Department of Veterinary Clinical Sciences, Jockey Club College of Veterinary Medicine and Life Sciences, City University of Hong Kong, Kowloon Tong, Hong Kong; 3 Centre for Animal Health and Welfare, City University of Hong Kong, Kowloon Tong, Hong Kong; 4 Storr Liver Centre, Westmead Clinical School and Westmead Institute for Medical Research, Faculty of Medicine and Health, The University of Sydney, Westmead, NSW 2145, Australia; 5 Sydney Institute for Infectious Diseases, University of Sydney at Westmead Hospital, Westmead, NSW 2145, Australia; 6 Department of Infectious Diseases and Public Health, Jockey Club College of Veterinary Medicine and Life Sciences, City University of Hong Kong, Kowloon Tong, Hong Kong

**Keywords:** cat, hepadnavirus, liver, hepatitis, viral, cancer, hepatitis B

## Abstract

Domestic cat hepadnavirus (DCH) is an emerging virus related to the hepatitis B virus (HBV). The pathogenic potential of DCH in cats remains to be established. The molecular prevalence of DCH varies widely in the regions investigated so far. The aim of this study was to determine the prevalence, load, and risk factors for DCH detection among cats in Hong Kong, and to generate molecular and epidemiological data on the DCH strains circulating in cats in Hong Kong. DCH DNA was detected using DCH-specific qPCR in 57/513 (11.1%) residual diagnostic blood samples from owned cats. The median viral load was 8.85 × 10^3^ copies/mL of whole blood (range for the 5th to the 95th percentile, 3.33 × 10^3^ to 2.2 × 10^5^ copies per mL). Two outliers had higher viral loads of 1.88 × 10^7^ copies/mL and 4.90 × 10^9^ copies/mL. DCH was detected in cats from 3 months to 19 years of age. Sex, age, neuter status, breed, or elevated serum alanine aminotransferase were not statistically associated with DCH DNA detection. On phylogenetic analysis based on 12 complete genome sequences, the Hong Kong DCH viruses clustered in Genotype A with viruses from Australia and Asia (clade A1), distinct from viruses from Europe (clade A2). Sequence analysis found that DCH has similar epsilon and direct repeat regions to human HBV, suggesting a conserved method of replication. Based on our findings, the DCH strains circulating in Hong Kong are a continuum of the Asiatic strains.

## 1. Introduction

Domestic cat hepadnavirus (DCH) is a newly discovered virus in the *Hepadnaviridae,* a family of over 17 species of partially double-stranded DNA viruses containing five genera of viruses infecting mammals (Genus *Orthohepadnavirus*), fish (Genus *Metahepadnavirus* and *Parahepadnavirus*), amphibians, reptiles (Genus *Herpetohepadnavirus*), and birds (Genus *Avihepadnavirus*) [1]. Orthohepadnaviruses cause varying degrees of liver pathology in their hosts. The prototype species, hepatitis B virus (HBV), is a major human pathogen. In 2019, an estimated 316 million people were living with chronic HBV infection [2], of whom >550,000 people died from liver cirrhosis and hepatocellular carcinoma (HCC) [3,4]. In woodchucks chronically infected with woodchuck hepatitis virus (WHV), hepatocellular carcinoma is almost inevitable with the lifetime risk approaching 100% [5].

Since DCH was discovered in a domestic cat in Australia in 2018 [6], the virus has been shown to be geographically widespread and common, with infected cats identified in Australia, New Zealand, Italy, the UK, Malaysia, Thailand, Japan, and the USA [6,7,8,9,10,11,12]. The reported prevalence of circulating DCH DNA varies from less than 1% in the USA and Japan to more than 18% in Thailand. This is likely an underestimate of the total number of DCH infections, since screening for serum DCH anti-core antibody (anti-DCHc, equivalent to the exposure marker anti-HBV core antibody for humans) identified 2.5 times as many cats exposed to DCH than were viraemic [13].

A potential role for DCH in feline liver disease is under investigation. Studies of feline liver biopsies reported an association between DCH DNA detection and hepatocellular carcinoma or hepatitis using cPCR, qPCR and in situ hybridization [14,15]. In addition, serological markers of liver disease have been identified as risk factors for DCH DNA positivity [7,9,15]. Other risk factors for DCH viraemia include, in some studies, coinfection with feline immunodeficiency virus (FIV) or feline leukaemia virus (FeLV), and a weak association with age > 2 years [6,7,8,9].

Investigations into the natural history and global epidemiology of DCH will assist in defining the potential threat to feline health from DCH infection as well as informing the comparative virology of hepadnaviruses. The aims of this study were to determine the presence, prevalence, and risk factors for DCH detection in owned cats, and to compare the genetic relatedness of DCH viruses in Hong Kong to those in other regions of the world.

## 2. Materials and Methods

### 2.1. Ethics Statement

This study was approved by the Animal Research Ethics Committee of the City University of Hong Kong (A-0150, 19 September 2020). 

### 2.2. Sample and Data Collection

Residual EDTA blood (*n* = 513) collected during diagnostic investigation of cats presenting to CityU Veterinary Medical Centre, Hong Kong between 1 June 2020 and 30 November 2020 was stored at −80 °C prior to DNA extraction. The patient’s age, sex, breed, neuter status, and serum ALT and total thyroxine (T4), if available, were recorded. Elevated T4 is diagnostic for hyperthyroidism, a common cause of elevation of ALT in cats > 8 years of age [16]. 

### 2.3. Sample Processing

DNA was extracted from 200 µL of whole blood and eluted in 50 µL using the QIAamp MinElute Virus Spin Kit (Qiagen, Germany). To confirm the presence of amplifiable DNA, conventional PCR (cPCR) for glyceraldehyde 3-phosphate dehydrogenase (GAPDH) was performed [17].

### 2.4. Real-Time Quantitative PCR (qPCR)

Real-time qPCR for DCH was performed as described previously [7]. For absolute DCH DNA quantification, a 1.4 kb-long fragment of the polymerase region of the Australian reference strain AUS/2016/Sydney was cloned using the TOPO XL-2 PCR cloning kit (Invitrogen, ThermoFisher Scientific, Waltham, MA, USA). Tenfold dilutions at 10^0^–10^9^ copies of plasmid per reaction were used to generate the standard curve. Standards and samples were run in triplicate. Ultrapure water and salmon sperm DNA were used as negative controls. Reactions were 25 μL, comprising 1 μL of neat template DNA plus 9 μL of water; or 10 μL plasmid standard and 15 μL of master mix, comprising IQ Supermix (Bio-Rad Laboratories SRL, Segrate, Italy), containing 0.6 μmol/L of each primer and 0.1 μmol/L of probe. Thermal cycling consisted of activation of Taq DNA polymerase at 95 °C for 3 min and 42 cycles of denaturation at 95 °C for 10 s and annealing-extension at 60 °C for 30 s. A sample was defined as positive if ≥10 copies of DCH DNA were detected in at least ≥ two of three replicates with a Ct value of ≥38.5. Thus, our PCR assay had a lower limit of quantification of 2500 copies/mL of blood. The cut-off for R-squared was 0.980 and for efficiency was 90–110%. 

### 2.5. Data Analysis

All data management and analyses were carried out using Stata v17 (StataCorp LLC, College Station, TX, USA). The univariable associations between DCH detection (positive/negative in qPCR) and each independent variable of interest (age, sex, neuter status, and breed) were evaluated using simple logistic regression models. Odd ratios (OR) and their corresponding 95% confidence intervals (CI) were estimated for each independent variable. Age was categorized into four groups: <6 months, 6 to 24 months, >24 to 84 months, and >84 months. Breed was defined as either mixed or purebred. The relationship between serum ALT level (as normal or elevated) and DCH detection was investigated using simple logistic regression in a subpopulation of cats for which serum ALT value was available, excluding those with elevated total T4 (*n* = 391). In addition, the correlation between DCH viral load and ALT level was assessed in cats with elevated ALT (*n* = 41) using Spearman’s rank correlation coefficient.

### 2.6. Whole Genome Sequencing

Samples testing positive for DCH DNA by qPCR were progressed to whole genome sequencing using a conventional PCR strategy with overlapping primers (Table 1) [6]. Whole genome sequences were obtained from a total of 12 cats, 2 cats from the epidemiological study and 10 other cats from Hong Kong for which no additional information was available.

### 2.7. Phylogenetic Analyses

Twelve complete genome sequences of DCH obtained from cats in Hong Kong were edited using Geneious Prime version 2021.2 (Biomatters Ltd., Auckland, New Zealand). High-quality sequences (>95%) were subjected to Basic Local Alignment Search Tool Nucleotide (BLASTN; https://blast.ncbi.nlm.nih.gov/ (accessed on 28 February 2022)) and FASTA Nucleotide (http://www.ebi.ac.uk/fasta33 (accessed on 28 February 2022)) searches using the default values to find homologous hits in the NCBI and EBI sequence databases, respectively. DCH sequences identified in this study were aligned with cognate DCH strains retrieved from the GenBank (NCBI) database in February 2022 by MAFFT [18] plugin, implemented in Geneious Prime version 2021.2 (Biomatters Ltd., Auckland, New Zealand). Substitution model settings for phylogenetic analysis and estimation of selection pressure on coding sequences were derived using “Find the best protein DNA/Protein Models”, implemented in MEGA X version 10.0.5 software [19,20]. The evolutionary history was inferred by using the maximum-likelihood method with the Hasegawa-Kishino-Yano model (two parameters) [21], a discrete gamma distribution to model evolutionary rate differences among sites (6 categories), and supplying statistical support with 1000 replicates. Phylogenetic analyses using other evolutionary models (Bayesian inference and neighbour-joining) were performed to compare the topology of the phylogenetic trees. Similar topologies with slight differences in bootstrap values at the nodes of the tree were observed. Accordingly, the maximum-likelihood tree was retained. Nucleotide sequences of strains HK_01 to HK_12 used for phylogeny were deposited in GenBank (NCBI) under accession nos. OP643851-OP643862, respectively.

### 2.8. Viral DNA Sequence Analyses

Sequence analysis was carried out on the consensus sequences of the HK DCH isolates and previously published DCH genomes. We included a human HBV DNA sequence (GenBank accession number U95551.1), DCH Clade A1 (NC040719.1, Sydney isolate), Clade B1 (MK117078.1, Italian isolate), and genotype B (LC685967.1, Rara isolate) sequences in the analysis as comparison groups. Genomes were aligned using Clustal Omega [22,23] and visualised using Jalview (v2.11.2.5) [24]. RNA structure of the epsilon regions was predicted using RNAstructure Web Server (v6.4) [25], using the MaxExpect algorithm. 

## 3. Results

### 3.1. Molecular Epidemiology of DCH in Cats in Hong Kong

All 513 samples tested positive for GAPDH and were available for study. There were similar proportions of male (55.0%) versus female cats (45.0%), and of mixed breed (47.4%) versus purebred cats (52.6%). Most cats (87.2%) had been neutered. The median age of the population was 115 months (ranging between 1.5 and 264 months). Population characteristics and results of the logistic regression analyses are presented in Table 2. 

DCH DNA was detected in 57/513 samples (11.1%). The median viral load was 8.85 × 10^3^ copies/mL of whole blood and the range for the 5th to the 95th percentile of this population was 3.33 × 10^3^ to 2.2 × 10^5^ copies per mL. Two cats had higher viral loads of 1.88 × 10^7^ copies/mL and 4.90 × 10^9^ copies/mL.

No independent variables (sex, neuter status, or breed) were statistically associated with DCH detection (Table 2). As all corresponding *p*-values were >0.2, multivariable modelling was not carried out. The DCH PCR-positive cats in the youngest age group were all 3 months of age. The oldest DCH positive cat was 19 years old. Among the 391 cats with serum ALT level available, 327 and 64 cats had normal (12–130 U/L) and elevated ALT (>130 U/L), respectively. Serum T4 measurement (normal) was available for 29 of 64 cats with elevated ALT. The prevalence of DCH detection in cats with elevated ALT (6/64, 7.8%) was not statistically different from the prevalence in cats with normal ALT (42/327, 12.8%) (Table 2). The Spearman’s correlation coefficient was −0.08 (*p* = 0.624), indicating no significant correlation between ALT levels and DCH viral load in DCH positive cats with elevated ALT.

### 3.2. Phylogenetic Analysis Positions Hong Kong DCH Isolates in Clade A1 with South-East Asian and Australian Strains

The complete 3184 bp genome sequence was obtained for 12 DCH viruses from domestic cats in Hong Kong (Table 3). The closest known relative to eleven isolates (HK_1-HK11) was the Malaysian DCH isolate MYS/2019/UPM CHV04 (MK902920), with 98.6–91.1% nucleotide identity at the full genome level. HK_12 had 98.8% nucleotide identity to the Australian virus, Sydney MH307930 (Table 3). 

**Figure 1:**
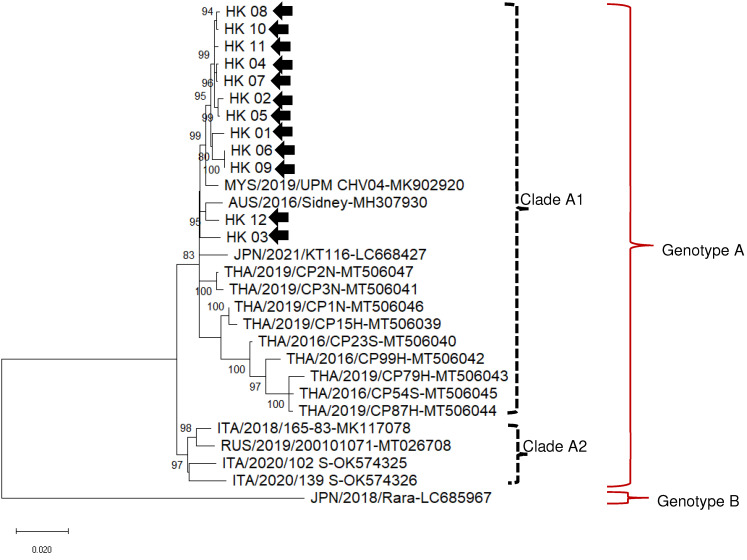
Unrooted phylogenetic tree based on the complete genomes of domestic cat hepadnavirus (DCH) viruses obtained in this study (HK01–HK12, black arrows) and reference strains retrieved from the GenBank database. Phylogeny was carried out using the Maximum Likelihood method and Hasegawa-Kishino-Yano model (two parameters), with a gamma distribution and invariable sites. The robustness of the individual nodes on the phylogenetic tree was estimated using 1000 bootstrap replicates. Bootstrap values greater than 70% are indicated. The subdivision in genotypes A and B, and clades A1 and A2 within genotype A are indicated. The scale bars indicate the estimated number of nucleotide substitutions per site.

On broader phylogenetic analysis (Figure 1) using whole genome nucleotide identity (Table 4), Hong Kong DCH viruses clustered with Malaysian, Thai, Australian, and European DCH viruses in a single genotype, defined as DCH genotype A. Ten Hong Kong DCH viruses clustered in a well-defined clade within genotype A, proposed as clade A1, forming a sister branch with Malaysian virus MYS/2019/UPM CHV04 (MK902920). Virus HK_12 clustered with the Australian 2016 Sydney DCH Virus (MH307930), forming a sister branch with HK_03. A second clade within genotype A, tentatively called clade A2contained DCH viruses sequenced from cats in Italy and Russia. The Japanese DCH virus, Rara, represents a separate genotype, proposed as DCH genotype B [12]. 

### 3.3. Sequence Analysis Shows Hong Kong DCH Isolates Have Conserved Features Common to Other DCH Isolates

We then performed an in-depth analysis of the 12 full-length DCH DNA sequences. Mutations (relative to the prototypic MH307930 Sydney isolate) were broadly distributed across the genome. Human HBV HBeAg-negative infections are linked with lower viral titres and commonly co-occur with mutations in the pre-core and basal core promoter regions. However, no mutations in putative pre-core or basal core promoter regions were observed in the DCH full-length sequences nor were any other mutations obviously associated with viral load.

**Figure 2:**
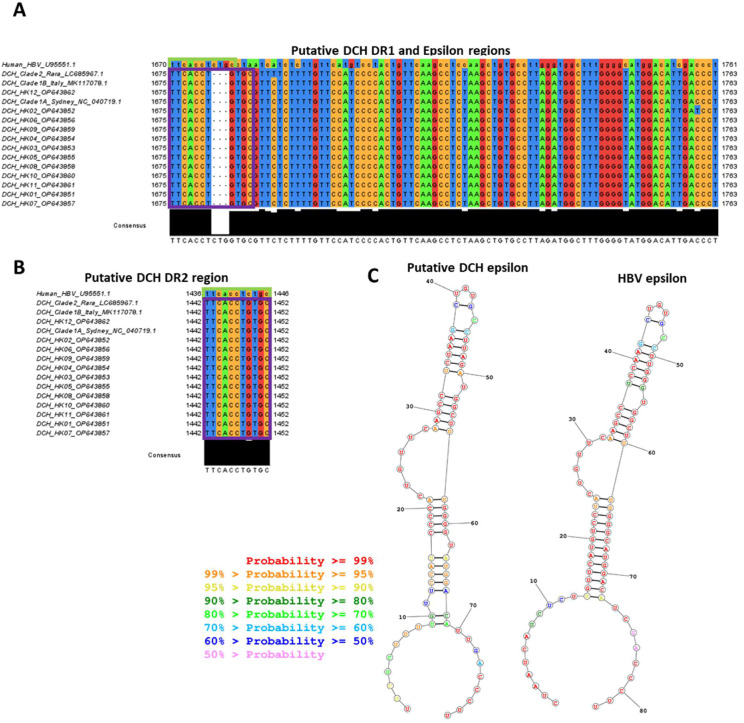
Sequence alignment and analysis of DCH isolates compared to human HBV sequences. The whole genomes of the HK DCH isolates were aligned to human HBV DNA sequence (GenBank accession number U95551.1), DCH genotype A, clade A1 (NC040719.1, Sydney isolate) and clade A2 (MK117078.1, Italian isolate), and genotype B (LC685967.1, Rara isolate) sequences in the analysis as comparison groups. DCH DR1 (**A**) and DR2 (**B**) sequences are boxed in purple and human HBV DR sequences are boxed in green. RNA structures of the putative DCH and HBV epsilon regions (**C**) were predicted by RNAstructure Web Server (v6.4) (Reuter and Mathews 2010), and calculated probability of binding is colour-coded according to the legend.

We identified putative DR1 (Figure 2A) and DR2 regions (Figure 2B), as well as a putative epsilon sequence that has strongly predicted stem loop structure almost identical to that of the human HBV epsilon (Figure 2C). These structures are required for hepadnavirus replication (in particular, initiating reverse transcription of the pre-genomic RNA) and are highly conserved in the stem-loops regions in all known isolates of DCH, including the HK DCH isolates. The short 3nt sequence exposed in the predicted bulge region (UUC, essential for initiating the priming of first-strand DNA synthesis) is identical in both DCH and human HBV, suggesting that DCH replicates in a similar manner to HBV. Confirming this, the 5′ end of the DCH DR sequence (TTC) where the self-primed polymerase should bind is conserved compared to human HBV, even though the 3′ remainder of the DR region is not.

## 4. Discussion

This study showed that DCH is circulating among cats in Hong Kong, with a molecular prevalence of 11.1%, as measured by DCH DNA detection in whole blood. The prevalence in other regions varies widely, with DCH-positive cats apparently uncommon in the USA (0.2%) [11], Japan (0.78%) [12], and UK (1.9%) [10]; whereas virus DNA has been readily detected in cats in Australia (6.5%) [6], Italy (10.8%; 4.2%) [7,26], Thailand (18.5%) [15], and Malaysia (12.3%) [9]. Despite study design differences, it is likely that there are genuine regional differences in DCH prevalence, potentially caused by various transmission dynamics, as with human HBV [27].

Our prevalence figures are likely an underestimate of DCH prevalence, as circulating DCH DNA does not identify all infected cats. PCR may not detect acute HBV infection (which is followed by viral clearance from serum), or phases of chronic infection where viral load may be below the detection limit. For human HBV, antibodies directed against HBV core protein, anti-HBc, appear during acute infection and IgG persists indefinitely as a marker of prior exposure [28]. An ELISA to detect anti-DCHc IgM or IgG using recombinant DCHc produced in a baculovirus-insect expression system has been developed [13]. Of 256 cats tested, 64 (25%) were positive for anti-DCHc while only 25 cats (9.8%) tested positive for DCH DNA, supporting our hypothesis that more cats have been exposed to DCH than those identified by PCR testing [13]. It is worth noting that another prototype anti-DCHc assay using recombinant His-tagged DCH core produced in *E. coli*, based on an anti-HBVc assay [29], was trialled (JB, VB, YR). Despite core self-assembly into virus-like particles on transmission electron microscopy, an ELISA using this antigen lacked sensitivity (data not shown). It seems possible that eukaryotic antigen expression of DCHc, as demonstrated by Fruci et al. [13], is required to produce antigen that can be recognized by feline antibodies produced in response to natural DCH infection.

In line with previous studies, neither breed (purebred versus mixed bred) nor sex was a risk factor for DCH detection in this Hong Kong cat population [7,8,9]. Similarly, we found that neuter status, not previously investigated as a risk factor, had no association with DCH detection. There was no association between age and DCH detection. The three youngest DCH positive cats were all 3 months of age, which is the age when maternal immunity is expected to wane. The greatest proportion of DCH positive cats was in the youngest age group here, consistent with the findings of three studies conducted in Italy [7,13,26]. These data show that vertical/perinatal transmission, the most common transmission route for human HBV, is possible for DCH. Moreover, owned-cats in Hong Kong are typically kept indoors; thus, future evaluation of free-roaming cats in the same geographical location is warranted, since transmission dynamics may well differ in this population.

No correlation was observed between serum ALT levels, a marker of hepatocellular injury, and DCH DNA detection, or virus load. In contrast, serum ALT was significantly associated with DCH DNA positivity in studies from Thailand and Malaysia [9,15]. A reported association between age >2 years and DCH detection [13] provided the rationale to exclude hyperthyroidism—a common cause of ALT elevation in cats > 8 years [16]—as a confounder in ALT analyses. ALT elevation occurs only transiently and during specific phases of chronic hepadnavirus infections, as these viruses are not directly cytopathic. Indeed, in human HBV infection, ALT can remain normal for decades after the initial infection. Thus, our observation in this study does not necessarily rule out a role for DCH in driving liver disease in cats.

If a pathogenic link between DCH and chronic hepatitis and hepatocellular carcinoma in cats can be established, insights into human HBV pathogenesis, which remains poorly characterized, may follow. Furthermore, a link between DCH and liver injury could justify the development of novel antivirals and vaccines for cats. Similarities between HBV and DCH DR/epsilon regions identified in this study, and the predicted viral receptor [11], support similar approaches to target DCH therapeutically. Unfortunately, many nucleoside/nucleotide analogues—the mainstay of human HBV antiviral treatment—are unacceptably toxic to cats [30]. Whether novel approaches to targeting HBV, such as capsid inhibitors or entry inhibitors, might be effective in cats, remains an unknown but exciting prospect. Detection of post-clearance neutralizing antibodies in cats (e.g., anti-DCH surface antibodies) could support development of a subunit vaccine, as is used in HBV prevention. Thus, this study contributes to the initial steps in understanding the links between DCH and cat health; further investigation into the clinical association and natural history of DCH should be a focus for the field.

Our analyses show that there is limited variation of DCH across large geographical regions. Phylogenetic analysis of complete DCH genome studies suggest isolates found in Hong Kong are closely related to those in Southeast Asia and Australia [8,11,12]. Using definitions stated previously for HBV, DCH exists as at least two genotypes (>8% nucleotide difference [31]), with all DCH viruses characterized so far clustering into a single genotype—referred to here as Genotype A—except for a single isolate from Japan, currently the only member of a second DCH genotype, Genotype B.

Given its ubiquitous maternal transmission and lack of cross-species tropism, human HBV and variations within its genome have been previously used to extrapolate the migration patterns of ancient human populations [32]. We have shown that DCH is likely to be similar in both regards, providing the tantalizing possibility that this feline virus too could be used to understand the temporal and geographical distribution of cats globally.

## 5. Conclusions

In conclusion, DCH was detected in approximately 1 in 10 owned cats in Hong Kong. Longitudinal studies of naturally infected cats undergoing comprehensive investigation for liver disease, combined with assessment of novel DCH markers, will shed light on DCH as a potential feline pathogen. It is likely that the pathogenesis associated with DCH occurs over many years to decades, providing a large window during which to consider novel therapeutics. Developing treatments would require broad stakeholder collaborations and long-term funding commitment but could offer key insights into human HBV pathogenesis. Most importantly, such research could provide direct and significant improvements to the lives of many cats and their owners.

## Figures and Tables

**Table 1:** Primer and cycling conditions used to obtain the full genomic sequence of domestic cat hepadnavirus.

Primer Set	Name	Primer Binding Site	Sequence	Product Size	Cycling Conditions		
		(Numbering Based on MH307930)			Denaturation	Annealing	Extension
Pair 1	184F_1	184–203	CCAACTTCCTGTCCTCCGAC	776	95 °C, 1 min	56 °C, 30 s	72 °C, 1 min
	959R_1	959–940	TCGATAAGCGGGGACAAAGG				
Pair 2	687F_2	687–706	ACAAAAACCAAACGCTGGGG	600	95 °C, 30 s	56 °C, 30 s	72 °C, 1 min
	1286R_2	1286–1267	AGGGACGTAGACGAAGGACA				
Pair 3 *	1107F_3	1107–1126	CCCCTTCCTATCCATGTCGC	802	95 °C, 1 min	58 °C, 30 s	72 °C, 1 min
	1908R_3	1908–1889	CCGTATGGTGAGGGGAACAG				
Pair 3b *	1107F_3	1107–1126	CCCCTTCCTATCCATGTCGC	1024	95 °C, 1 min	58 °C, 30 s	72 °C, 1 min
	Cir5R	2130–2113	TGCGAATCCAGGTGCCAA				
P3Euro *	P3Euro2F	1099–1118	TCCATTCACCCCTTCCTATC	790	95 °C, 1 min	55 °C, 30 s	72 °C, 1 min
	P3Euro3R	1888–1868	TGTTCCCTACCTGTAAGTTCC				
Pair 4	1729F_4	1729–1748	GCCTTAGATGGCTTTGGGGT	768	95 °C, 30 s	56 °C, 30 s	72 °C, 1 min
	2496R_4	2496–2477	CTTCAGACGGCGGAGTTCAT				
Pair 5	2110F_5	2110–2129	CTTTTGGCACCTGGATTCGC	852	95 °C, 30 s	56 °C, 30 s	72 °C, 1 min
	2952R_5	2961–2942	TTGGTGATTGGTTGAGCGGA				
Pair 6	2752F_6	2752–2771	GGGGTTTTTCCCTCGTCACA	1061	95 °C, 30 s	56 °C, 30 s	72 °C, 1 min
	Cir4R	625–605	AGATGTTCCACACTCTTAGCC				

* Primer pairs 3, 3b, and 3Euro were used in succession, if required, until a product was obtained.

**Table 2:** Univariable associations between domestic cat hepadnavirus DNA PCR result and independent variables in owned cats from Hong Kong using simple logistic regression.

Variable	Categories	PCR Positive	PCR Negative	% PCR Positive	Total	OR	95% CI	*p*-Value
Age (months)	<6	3	17	15.0	20	-	-	0.246 *
	6–24	2	50	3.8	52	0.23	0.03–1.47	0.120
	>24–84	13	103	11.2	116	0.71	0.18–2.78	0.628
	>84	39	286	12.0	325	0.77	0.22–2.76	0.691
Sex	Female	24	206	10.4	230	-	-	-
	Male	33	250	11.7	283	1.13	0.65–1.98	0.661
Neutered	Yes	48	379	11.2	427	-	-	-
	No	8	56	12.5	64	1.13	0.51–2.51	0.768
	Unknown	-	-	-	22	-	-	-
Breed	Mixed	26	216	10.7	242	-	-	-
	Pure	31	240	11.4	271	1.07	0.62–1.86	0.802
Serum ALT (U/L)	Normal (12–130)	42	285	12.8	327	-	-	-
	High (>130)	5	59	7.8	64	0.57	0.22–1.51	0.263
	Unknown	-	-	-	122	-	-	-

* Overall *p*-value for ‘age’. Abbreviations: OR, odd ratio; CI, confidence interval; - = intentionally blank.

**Table 3:** Genomic features of complete genomes of the domestic cat hepadnavirus (DCH) strains sequenced in this study.

Sample ID	Accession	Size (nt)	L Region		S Region		X Region		C Region		Identity to Reference Sequences *	
			nt	aa	nt	aa	nt	aa	nt	aa	DCH Strain ** (Accession nr.)	nt Identity %
HK_01	OP643851	3184	2514	838	1146	382	438	146	657	219	MYS/2019/UPM CHV04 (MK902920)	98.9
HK_02	OP643852	3184	2514	838	1146	382	438	146	654	218	MYS/2019/UPM CHV04 (MK902920)	98.9
HK_03	OP643853	3184	2514	838	1146	382	438	146	657	219	MYS/2019/UPM CHV04 (MK902920)	98.6
HK_04	OP643854	3184	2514	838	1146	382	438	146	657	219	MYS/2019/UPM CHV04 (MK902920)	99.1
HK_05	OP643855	3184	2514	838	1146	382	438	146	657	219	MYS/2019/UPM CHV04 (MK902920)	99.0
HK_06	OP643856	3184	2514	838	1146	382	438	146	657	219	MYS/2019/UPM CHV04 (MK902920)	98.8
HK_07	OP643857	3184	2514	838	1146	382	438	146	657	219	MYS/2019/UPM CHV04 (MK902920)	99.1
HK_08	OP643858	3184	2514	838	1146	382	438	146	657	219	MYS/2019/UPM CHV04 (MK902920)	99.1
HK_09	OP643859	3184	2514	838	1146	382	438	146	657	219	MYS/2019/UPM CHV04 (MK902920)	98.8
HK_10	OP643860	3184	2514	838	1146	382	438	146	657	219	MYS/2019/UPM CHV04 (MK902920)	99.1
HK_11	OP643861	3184	2514	838	1146	382	438	146	657	219	MYS/2019/UPM CHV04 (MK902920)	99.1
HK_12	OP643862	3184	2514	838	1146	382	438	146	657	219	AUS/2016/Sydney (MH307930)	98.8

* Identity based on the full genome sequence; ** genome sequence with the highest identity on interrogation of GenBank database with BLAST; L, polymerase; S, surface; C, core; nt, nucleotides; aa, amino acids.

**Table 4:** Nucleotide identity between domestic cat hepadnavirus (DCH) Genotypes and Clades based on the overall genome.

		Genotype A		Genotype B
		clade A1	clade A2	
**Genotype A**	clade A1	100	95.8–97.5	88.7–89.2
	clade A2	95.8–97.5	100	89.0–89.5
**Genotype B**		88.7–89.2	89.0–89.5	100
